# Effect of Annealing in Magnetic Field on Ferromagnetic Nanoparticle Formation in Cu-Al-Mn Alloy with Induced Martensite Transformation

**DOI:** 10.1186/s11671-016-1453-2

**Published:** 2016-05-03

**Authors:** Anatoliy Titenko, Lesya Demchenko

**Affiliations:** Institute of Magnetism of National Academy of Sciences and Ministry of Education and Science of Ukraine, 36-b Vernadsky Boulevard, Kyiv, 03142 Ukraine; Department of Physics of Metals, National Technical University of Ukraine “Kyiv Polytechnic Institute”, 37 Peremohy Avenue, Kyiv, 03056 Ukraine

**Keywords:** Martensite transformation, Cu-Al-Mn alloy, Aging, Annealing, Magnetic field, Ferromagnetic nanoparticles, 81.30.Kf, 81.40.Ef, 81.40.Jj, 62.20.-x

## Abstract

The paper considers the influence of aging of high-temperature phase on subsequent martensitic transformation in Cu-Al-Mn alloy. The morphology of behavior of martensitic transformation as a result of alloy aging under annealing in a constant magnetic field with different sample orientation relatively to the field direction and without field was studied for direct control of the processes of martensite induction at cooling. Temperature dependences of electrical resistance, magnetic susceptibility, and magnetization, as well as field dependences of magnetization, and phase composition were found. The tendency to the oriented growth of precipitated ferromagnetic phase nanoparticles in a direction of applied field and to an increase of their volume fraction under thermal magnetic treatment of material that favors a reversibility of induced martensitic transformation is observed.

## Background

Phase transformations of martensitic type are inherent to a wide class of materials and alloys, which are characterized by structural features that determine peculiarities of their formation and physical properties. Priority in research of new materials belongs to alloys, in which the structural phase transformations of martensitic-type are realized in a ferromagnetic matrix, such as Heusler alloys, intermetallic compounds Co-Ni-Al, Co-Ni-Ga alloys, and Fe-based alloys.

A mechanism of the behavior of martensitic transformation (MT), occurring in alloy after decomposition of solid solutions with precipitation of ferromagnetic nanoparticles in the nonferromagnetic matrix, is not less attractive. Such behavior is typical for Cu-Co, Cu-Ni-Fe, Cu-Ni-Co, and Ni-Mn alloys. In ternary Cu-Al-Mn Heusler alloy, MT can take place [1] and an appearance of long-range ferromagnetic order in a system of superparamagnetic nanoparticles, dissolved in the nonmagnetic matrix, is caused by the cooperative ordering of their magnetic moments. Alloys of this system demonstrate high values of characteristics of shape memory effects and superelasticity [2–4]; they also exhibit a giant magnetoresistance [5].

As it has been established by neutron diffraction measurements [6] that only the Mn atoms possess a magnetic moment, Winkler et al. [7] have shown that the chemical order determines the magnetic behavior of the alloys because it establishes the distances between Mn atom pairs. The Mn-Mn interactions can be ferromagnetic as in Cu_2_MnAl or antiferromagnetic as in MnO, or a mixture of both as in Cu-Mn alloys [6], depending on the magnetic moment of each atom and the distances between them. Only 35 % of Mn atoms are involved in the ferromagnetic Cu_2_MnAl phase formation while others are dissolved in the nonmagnetic matrix [8].

The stoichiometric compound CuAlMn is ferromagnetic with a Curie temperature of 630 K, and it is known that the entire magnetic moment of the system must be attributed to the Mn atoms [9] coupled via the Ruderman-Kittel-Kasuya-Yosida interaction [10]. After a suitable heat treatment [11], the Cu_3−*x*_AlMn_*x*_ (*x* < 1) alloys display an L2_1_ structure (Fm3m space group) that can be viewed as defined on a bcc lattice divided into four interpenetrated fcc sublattices.

The high-tempetarure β_1_-phase of binary and ternary copper-aluminum alloys has an ordered bcc structure of DO_3_ type [12]. The miscibility gap is found below the L2_1_ ordering line in Cu-Al-Mn systems. Phase separation between nonmagnetic DO_3_(Cu_3_Al)-rich and ferromagnetic L2_1_(Cu_2_AlMn)-rich domains takes place in this region [13].

Martensitic phases, formed in alloys of various compositions, are closely-packed structures, which differ by the order of stacking of closely-packed planes: β_1_′(3R) or γ′(2H) [12]. The martensite crystals inherit coherent particles from austenite, the crystal lattice of which varies due to an elastic deformation that is caused by changing conditions of lattice conjugation of nanoparticles and a matrix at MT.

Annealing in the β phase produces changes in the martensitic transformation temperature. These changes have been shown to be associated with order-disorder process at low temperature [14] or precipitation of equilibrium phases at higher temperatures [15].

Thus, by varying regimes of aging of high-temperature phase (austenite), it is possible to considerably affect the process of its decomposition [16] that can result in significant changes of characteristic temperatures and a hysteresis of MT in Cu-Al-Mn alloys. By following certain conditions in aging process, it is possible to achieve a situation that results in an increase of MT start temperature (*M*_s_) and a decrease of MT temperature hysteresis (Δ*T* = *A*_f_ − *M*_s_, where *A*_f_ is a final temperature of inverse transition, *M*_s_ is a start temperature of direct transformation). The first condition is related to β_1_–matrix depletion with manganese, and the second one is associated with a decrease in the shear strain of transformation area due to a reduction of elastic energy (*E*_el_) on phase interface. A certain heat treatment allows controlling a number and a size of particles in the alloy, and MT characteristic temperature and hysteresis value substantially depend on the size and amount of particles [16]. Recent studies [17, 18] have shown that Cu-Mn-Al alloys with a high content of Mn have acceptable mechanical properties that open the possibilities of their practical application.

To develop a concept of the nature and character of MT behavior and to directly control the process of MT induction, it is of great interest to study the morphology of MT behavior in Cu-Al-Mn alloy after an aging of high-temperature phase as a result of annealing in a constant magnetic field depending on sample orientation relative to the field. For this purpose, in order to select the best regime of thermal or thermal-magnetic treatment, the Cu-Al-Mn alloy with a chemical composition which provides the lowest value of MT temperature hysteresis was chosen for the investigations.

## Methods

The Cu-Al-Mn alloy was smelted in an induction furnace in an argon atmosphere. According to energy dispersive X-ray fluorescent analysis data, the chemical composition of the alloy is as follows (in mass %): Cu (84.7), Al (11.1), and Mn (4.2) with error in margin ±0.5 %.

**Fig. 1:**
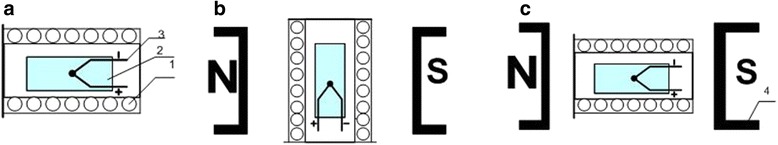
Scheme of placement of Cu-Al-Mn alloy samples at annealing: **a** without a field; **b** in the magnetic field perpendicular to the main axis of sample; and **c** in the magnetic field parallel to the main axis of sample, where 1 is an electrical heater, 2 is a sample, 3 is a thermocouple, and 4 is а magnetic pole

After homogenizing annealing at 1123 K for 10 h, the samples were quenched in water and then were annealed at a constant temperature of 498 K for 3 h. The permanent magnetic field with a strength of 1.5 kOe was created by the selection of ferromagnetic plates which were spaced by a distance, required for a placement of an electrical heater with a sample in their gap. The placement of samples in relation to a direction of magnetic field and without it is shown in Fig. 1. To eliminate the influence of magnetic field of the electrical heater on a sample, the bifilar nichrome wire winding was used. The samples had a working length of 20 mm with a cross section of 2 × 2 mm^2^.

Characteristic temperatures and MT hysteresis were determined from the curves of temperature dependences of electrical resistance (*ρ*/*ρ*_mах_) and magnetic susceptibility (*χ*/*χ*_mах_) according to a standard technique. Temperature and field dependences of magnetization were also a subject of study. Phase composition of samples was defined using X-ray diffractometer Rigaku Ultima IV in monochromatic К_α_-radiation of Cu-anode.

## Results and discussion

**Fig. 2:**
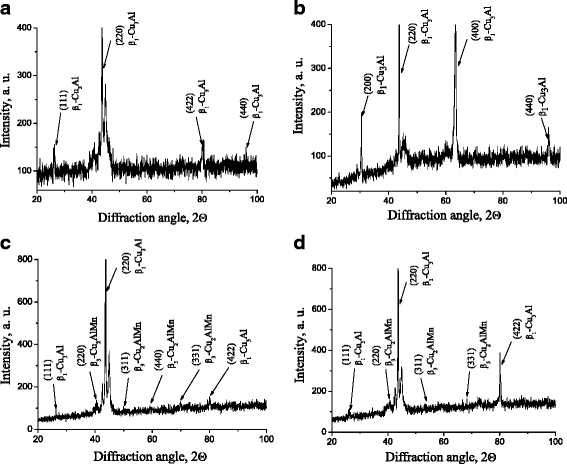
X-ray diffraction patterns of Cu-Al-Mn alloy samples: **a** after quenching in water; **b** after annealing without a magnetic field; **с** after annealing in the magnetic field perpendicular to the main axis of a sample; and **d** after annealing in the magnetic field parallel to the main axis of a sample

The results of X-ray phase analysis (Fig. 2a) testify the β_1_-phase (of close to Cu_3_Al composition) formation after the cooling (with use quenching in water) as a result of ordering (β → β_1_) of high-temperature (austenitic) β-phase. The subsequent annealing leads to the Cu-Al-Mn alloy aging accompanying by precipitation of dispersed particles of ferromagnetic β_3_-phase of Cu_2_AlMn composition in β_1_-matrix [15, 16].

After annealing without magnetic field (Fig. 2b), there are no reflections observed from β_3_-phase on diffraction patterns that can be explained by a low percentage of this phase in the alloy or/and its high dispersed (nanosized) state.

The annealing in the magnetic field (Fig. 2c, d) favors the precipitation of β_3_-phase (Cu_2_AlMn) particles in β_1_-matrix, as argued by an appearance of reflections of weak intensity from β_3_-phase at appropriate angles. The weak intensity of reflections can indicate that the particles of precipitated phase are nanosized. Change in sample orientation relative to the magnetic field direction during the aging affects β_3_-phase formation. After annealing in the parallel field, the diffraction lines from β_3_-phase are slightly more intense than after annealing in the perpendicular field that can testify about more amount of this phase in the alloy.

**Fig. 3:**
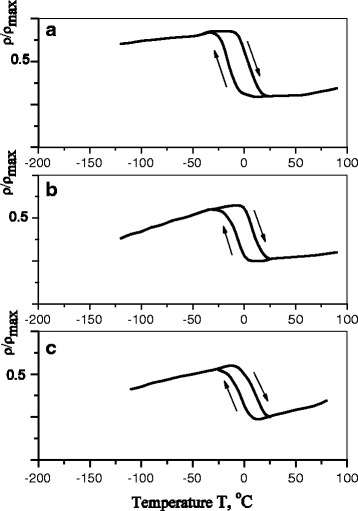
Temperature dependence of electrical resistance of Cu-Al-Mn alloy samples after aging at different regimes: **a** without a field; **b** in the perpendicular magnetic field; and **c** in the parallel magnetic field

The character of the temperature dependence of electrical resistance is the same like in metal alloys (Fig. 3). At the temperature of MT start (*M*_s_), the growth of electrical resistance of samples, associated with the replacement of a volume fraction of initial phase by the phase which has a more defect structure, occurs. As it is evident from Fig. 3, further heating of samples after MT has a reversible character with the inverse sequence of reproduction.

**Fig. 4:**
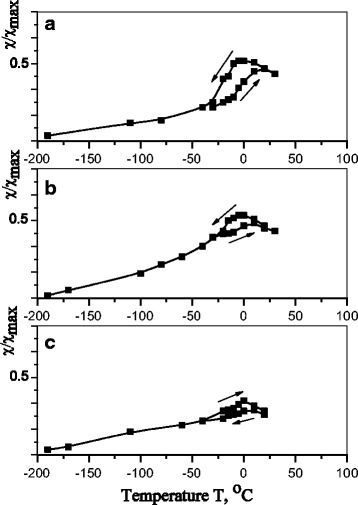
Temperature dependence of low-field magnetic susceptibility of Cu-Al-Mn alloy samples after aging at different regimes: **a** without a field; **b** in the perpendicular magnetic field; and **c** in the parallel magnetic field; an *arrow* indicates heating-cooling

According to the results of measurements of low-field magnetic susceptibility (*χ*/*χ*_mах_) of Cu-Al-Mn alloy at different regimes of aging (see Fig. 4), there is a slight increase in the start temperature (*M*_s_) of direct β_1_↔β′ transformation after annealing in magnetic field and a decrease in the width of MT temperature hysteresis (Δ*T*). The increase in M_s_, in this case, is caused by depletion of β_1_-matrix with manganese. A lower amplitude of a transition jump on the *χ*/*χ*_mах_ curves for samples with thermomagnetic treatment is associated with a dominant orientation of nanoparticles which are allocated in a direction of applied external magnetic field. In the case of thermal treatment without a magnetic field, the spherical concentration inhomogeneities are distributed in a sample, mainly, chaotically, in contrast to thermomagnetic treatment, which orders the Cu_2_MnAl phase nanoparticles nucleation.

Such effect of thermal magnetic treatment on magnetic susceptibility can be explained by the fact that magnetic characteristics of the investigated alloy are defined by an interaction of nanoparticles of precipitated phase. Nanoparticles that are within the martensite crystals are subjected to considerable coherent stresses caused by a crystal lattices mismatch of martensite and nanoparticles, which leads to magnetic anisotropy of nanoparticles ensemble. The crystal lattice mismatch sharply increases when cubic lattice of the austenitic matrix is transformed into the orthorhombic lattice of martensite, and, thus, the temperature dependence of magnetic susceptibility is caused by a corresponding change in magnetic anisotropy of nanoparticles.

As it is known from the phenomenological theory of diffusion decay [19], the change in free energy of the system is represented as three components, which are determined by the change in volume, surface, and elastic energies:

$$ \varDelta G = \varDelta {G}_{\mathrm{v}} + \varDelta {G}_{\mathrm{s}} + \varDelta {G}_{\mathrm{g}}. $$

When the magnetic field is applied during the alloy aging, the phase, which forms, can have a superparamagnetic state [20], and, in this case, a component, responsible for an interaction of magnetization of phase with the external magnetic field (Δ*G*_m_), is added to the expression for free energy of system that leads to the equation correction:

$$ \varDelta G = \varDelta {G}_{\mathrm{v}} + \varDelta {G}_{\mathrm{s}} + \varDelta {G}_{\mathrm{g}}+\varDelta {G}_{\mathrm{m}}. $$

The radius of a critical nucleus of a new phase, which can be defined using a standard procedure of free energy minimization relative to the radius of new phase nucleus, is as follows [21]:

1 $$ {R}_{\mathrm{c}}={\gamma}_{\mathrm{s}}/\left(IB+{g}_{\mathrm{v}}-{e}_{\mathrm{v}}\right), $$

where *γ*_s_ is a surface energy of the system, *g*_v_ is a specific change in thermodynamic potential of precipitated phase relative to the matrix, *е*_v_ is a specific elastic energy, *I* is a magnetization of a new phase, and *B* is an induction of magnetic field. So, the energy of formation of a new phase critical nucleus, in turn, can be expressed by a formula [21]:

2 $$ \varDelta {G}_{\mathrm{c}}=16\pi {\gamma}_{\mathrm{s}}^3/3\left(IB+{g}_{\mathrm{v}}-{e}_{\mathrm{v}}\right) $$

**Fig. 5:**
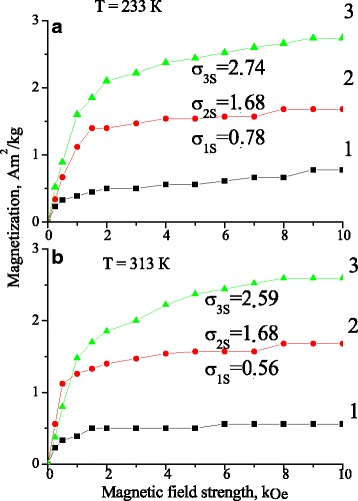
The field dependences of magnetization of Cu-Al-Mn alloy samples after aging at different regimes: (*1*) without a magnetic field; (*2*) in the perpendicular magnetic field; (*3*) in the parallel magnetic field; **a** in martensite state (−40 °C); and **b** in austenite state (+40 °C); where *σ*_*s*_ is a saturation magnetization

**Fig. 6:**
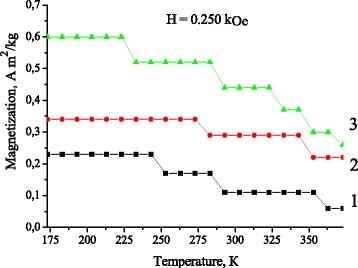
The temperature dependences of magnetization of Cu-Al-Mn alloy samples after aging at different regimes: (*1*) without a magnetic field; (*2*) in the perpendicular magnetic field; and (*3*) in the parallel magnetic field

It should be noted that in the case of applying an external magnetic field, the critical nucleus size and the energy of its formation decreases. At a certain temperature and aging time, the application of magnetic field stimulates the formation of new nuclei, which agrees well with the behavior of magnetization (Figs. 5 and 6), which, in turn, depends on the amount of precipitated ferromagnetic nanoparticles. A magnetic moment of alloy depends on, as it is known, the magnetic moment of manganese atoms, and the distance between them as well [6].

According to the Boltzmann distribution, the magnetization of an ensemble of non-interacting ferromagnetic nanoparticles is defined by a relation [20, 22]:

3 $$ I=nmL\left( mH/kT\right), $$

where *L* is the Langevin function equal to:

4 $$ L\left( mH/kT\right)=\operatorname{cth}\left( mH/kT\right)-kT/ mH, $$

where *m* is a magnetic moment of the nanoparticle, *n* is a number of nanoparticles per unit volume, and *k* is the Boltzmann constant.

By applying an external magnetic field, the particles growth in the direction of field takes place due to the minimization of magnetostatic and surface energies of particles resulting in a system free energy decrease. When choosing an effective temperature interval of thermal magnetic treatment (TMT) for aging, it was taken into account the fact that the interfacial energy and magnetization possess low values near a decay curve on a diagram of states [23], so, the TMT for the formation of anisotropic ferromagnetic nanoparticles was carried out in region of these temperatures (at 225 °C).

As it follows from Figs. 4, 5 and 6 a number of precipitated nanoparticles are maximal in the case of annealing in the parallel magnetic field. In turn, an increase in a number of precipitated nanoparticles stimulates the growth of start temperature of direct MT and the reduction of MT hysteresis. The transformation hysteresis is small, thus, an accurate determination of the equilibrium temperature *T*_0_ between the β and martensitic phase as *T*_0_ ≈ (*M*_s_ + *A*_f_)/2 becomes possible [24].

Reducing the MT temperature hysteresis (Δ*Т*), in this case, agrees well with results of [16]:

5 $$ \varDelta T/{T}_0={E}_{\mathrm{m}}\left(1-\alpha \right)/L, $$

where *T*_0_ is the temperature of equilibrium between austenitic and martensitic phases, *L* is the MT heat, and *α* is a coefficient which depends on strength characteristics of the material and determines the degree of coherent stress relaxation.

A low cold deformation reduces the magnitude of the elastic modulus of solid solutions (as well as pure metals). At high degrees of deformation, it is possible to increase it due to the formation of texture. The presence of magnetostriction phenomenon (connected with a change in linear size at magnetic ordering) in ferromagnetics leads to the fact that they have a lower modulus of elasticity: *E*_ferr_ = *E*_norm_ − *E*. The increase of volume fraction of precipitated phase nanoparticles leads to decrease in a strain of the transformation, and, as a result, to the reduction of the system elastic energy (*E*_el_), what stimulates the reducing the MT temperature hysteresis [16].

## Conclusions

The paper was attempted to the process control of phase formation at the aging of high-temperature phase using a constant magnetic field with the strength of 1.5 kOe in order to optimize the parameters of martensite transformation behavior in Cu-Al-Mn alloy. With the help of the phenomenological theory of diffusion decay and obtained experimental results, the behavior of induced MT in Cu-Al-Mn alloy was explained and its causes were found out. One can assume that applying a magnetic field stimulates the formation of ferromagnetic nanoparticles during the decay of solid solution. In addition, thermomagnetic treatment helps to create effective nucleation centers of nanoparticles in Cu-Al-Mn alloy during the aging of high-temperature phase.

Thus, annealing in a magnetic field promotes the increasing of ferromagnetic nanoparticles number in the nonferromagnetic matrix, as evidenced by measuring the magnetic properties of the material sample, such as field dependences of magnetization and low-field magnetic susceptibility.

For the further and in-depth understanding of decay processes impact on the behavior of martensitic transformation, it is planned to continue the work in the direction of the study of microstructural features of phase formation by applying direct methods.

## Abbreviations

MT: martensitic transformation
TMT: thermal magnetic treatment
